# Prolonged Duration of Hashitoxicosis in a Patient with Hashimoto’s Thyroiditis: A Case Report and Review of Literature

**DOI:** 10.7759/cureus.2804

**Published:** 2018-06-14

**Authors:** Amir Shahbaz, Kashif Aziz, Muhammad Umair, Issac Sachmechi

**Affiliations:** 1 Internal Medicine, Icahn School of Medicine at Mount Sinai Queen Hospital Center, New York, USA

**Keywords:** hashimoto's thyroiditis, hashitoxicosis, grave's disease

## Abstract

Hashitoxicosis is the initial hyperthyroid phase of patients with Hashimoto's thyroiditis and, usually, this phase lasts for one to two months. We report a case of a 21-year-old male who had Hashitoxicosis of two years duration before converting to Hashimoto’s hypothyroidism. He initially presented with complaints of increased appetite, heat intolerance, fatigue, and sweating. On a physical exam, he had mild exophthalmos with lid lag and a fine tremor in the hands. Thyroid function tests also confirmed that the patient had hyperthyroidism. Thyroglobulin antibody and thyroid peroxidase antibody were both positive. He also had mildly elevated thyroid-stimulating immunoglobulin (TSI) but decreased radioactive iodine uptake scan. Based on the clinical presentation and biochemical test, a diagnosis of Hashitoxicosis was made. This hyperthyroid phase lasted for a period of two years. The patient eventually developed hypothyroidism suggesting that Hashimoto's thyroiditis was the most likely diagnosis. He was started on levothyroxine replacement therapy and remained euthyroid on levothyroxine since that day. The initial presentation mimicked Grave’s disease, but with decreased radioiodine uptake, despite the high TSI level, leading us to treat him medically and not with radioactive iodine therapy. The patient was thus spared unnecessary radioactive iodine therapy* *(RAI) therapy.

## Introduction

Hashimoto thyroiditis is a chronic autoimmune disease that involves gradual thyroid failure and is present with or without goiter formation. Hashitoxicosis is the hyperthyroid phase of Hashimoto’s thyroiditis. It is caused by the destruction of the thyroid follicles by an inflammatory process that releases preformed thyroid hormones into the serum [1]. Hashimoto thyroiditis is characterized by elevated titers of antibodies like anti-thyroglobulin (anti-TG) and/or anti-thyroid peroxidase (anti-TPO) antibody. Hashitoxicosis is differentiated from Graves' disease by scarce radioiodine uptake by the thyroid gland [2]. We present a case of unusual prolong duration of Hashitoxicosis in a patient with Hashimoto’s thyroiditis. This case reflects the importance of the continued follow-up of patients with autoimmune thyroid disease.

## Case presentation

A 21-year-old male presented with a history of increased appetite, heat intolerance, fatigue, and sweating. On physical examination, he appeared to be anxious, He had a sinus rhythm with a heart rate of 96/min. His blood pressure was 126/85 mmHg. He also had mild exophthalmos with lid lag and a fine tremor on outstretching of the hands. Thyroid stimulating hormone (TSH) was suppressed 0.02 m IU/ml (0.04-4.50) while free thyroxine (free T4) was 2 ng/ml (0.8- 1.8) was elevated. The suppressed TSH and elevated free T 4 was consistent with hyperthyroidism. Anti-TG and anti-TPO were 517 IU/ml (<20 IU) and > 1,000 IU/ml (<35 IU/ml), respectively. He also had mildly elevated thyroid stimulating immunoglobulin (TSI): 164.9 (<125), but his radioactive iodine uptake scan was 9.6 (normal 9 5% to 30%). Based on the clinical presentation and biochemical tests, a diagnosis of hyperthyroidism was made. Because of the severity of symptoms, methimazole and atenolol were initiated to treat hyperthyroidism. The TSH level gradually increased to a high normal level over 16 months, after which the dose of methimazole was gradually decreased. Methimazole was finally discontinued after two years. On a subsequent follow-up visit, the TSH level increased to 4.15 mIU/ml, suggesting subclinical hypothyroidism. Eventually, after seven months, he presented with fatigue and weight gain and was found to have high TSH of 13 mIU/ml and low free T4 of 0.9 ng/ml, suggesting hypothyroid, with Hashimoto's thyroiditis as the most likely diagnosis. He was started on levothyroxine replacement therapy and remained euthyroid on levothyroxine since that day. The initial presentation mimics Grave’s disease, but his normal radioiodine uptake, despite the high TSI level, led us to treat him medically and not with radioactive iodine (RAI) therapy.

## Discussion

Chronic autoimmune thyroiditis is the most common cause of hypothyroidism. It is characterized by elevated levels of thyroid antibodies and decreased radioiodine uptake by the thyroid gland. Anti-TPO antibodies are present in about 90% of the patients with Hashimoto’s thyroiditis, and anti-TG antibodies are positive in about 60% of the patients with chronic thyroiditis [1]. Hashimoto’s thyroiditis usually presents as subclinical or overt hypothyroidism. Rarely, a patient has the signs and symptoms of hyperthyroidism at the initial presentation, which resolve in a few weeks to months. This hyperthyroid phase is followed by the euthyroid or hypothyroid state [2]. Hashitoxicosis is the initial hyperthyroid phase in chronic autoimmune thyroiditis. It occurs due to the release of preformed thyroid hormones from the inflamed thyroid gland [3]. The presentation in our patient was consistent with Hashitoxicosis. Hashitoxicosis is usually treated symptomatically but due to the prominent signs and symptoms of hyperthyroidism, we started antithyroid medications in our patient. On subsequent follow-up visits, the patient gradually returned to the baseline over a period of two years. The prolonged duration of the hyperthyroid phase in our patient was unusual.

Nabhan et al. found that eight of 69 patients with Hashimoto thyroiditis (11.5%) initially presented with hyperthyroidism. The duration of hyperthyroidism ranged from one to five months and positively correlated with thyroid peroxidase autoantibody levels at presentation. Three patients were diagnosed with hypothyroidism after an average of 46.3 ± 13.2 days and five patients were diagnosed with euthyroid after an average of 112.8 ± 59.8 days [4]. Wasniewska et al. investigated the outcome of Hashitoxicosis outcome in 14 children. Due to a more severe presentation, four patients required methimazole. A definitive resolution of hyperthyroidism was recorded 8.3 ± 6.3 months after the diagnosis [5]. The presence of thyroid stimulation antibodies in our patient was intriguing. It may be due to the fact that both Hashimoto’s thyroiditis and Graves’s disease are manifestations of the same disease spectrum. In Hashimoto thyroiditis, as well as in Graves’ disease, thyroid-reactive T lymphocytes are formed and infiltrate the thyroid gland [6]. A linkage analysis of thyroid antibody production shows that both Grave’s disease and Hashimoto thyroiditis represent a distinct, but related, phenotype due to an overlap in antibody production [7]. One possible explanation of the unusually long duration of hyperthyroidism in our patient was the presence of these stimulating antibodies apart from the destruction of thyroid follicles [8]. Treatment with antithyroid medications, radioactive iodine(131I), and surgery are appropriate only for hyperthyroidism due to Grave’s disease and not for other forms of thyrotoxicosis [9]. The patient was followed for two years. Correction of the hyperthyroid state was achieved after 16 months of therapy, and the anti-thyroid treatment gradually stopped. After that, on follow-up visits, the patient finally presented with symptoms of overt hypothyroidism, requiring levothyroxine. This case represents an example of an unusually prolonged phase of Hashitoxicosis in a patient with Hashimoto’s thyroiditis, which is an uncommon, yet important, cause of hyperthyroidism. A significant overlap of symptoms with Graves’ disease makes it a diagnostic challenge to differentiate between the two diseases. A careful and detailed evaluation of each patient is necessary to avoid unnecessary RAI therapy.

## Conclusions

This case is an example of an unusually prolonged phase of Hashitoxicosis in Hashimoto’s thyroiditis. We recommend a long-term, regular, follow-up of patients with Hhashitoxicosis to avoid RAI therapy.
